# Pneumonia vaccination timing in relation to starting conventional synthetic disease-modifying antirheumatic drugs in patients with rheumatoid arthritis

**DOI:** 10.1136/annrheumdis-2020-217255

**Published:** 2020-06-26

**Authors:** Ruth E Costello, Jenny H Humphreys, Kevin L Winthrop, William G Dixon

**Affiliations:** 1 Centre for Epidemiology Versus Arthritis, The University of Manchester, Manchester, Manchester, UK; 2 Division of Infectious Diseases, Oregon Health & Science University, Portland, Oregon, USA; 3 Department of Rheumatology, Salford Royal NHS Foundation Trust, Salford, UK

**Keywords:** rheumatoid arthritis, vaccination, DMARDs (synthetic)

Patients with rheumatoid arthritis (RA) are at increased risk of infections, and pneumococcal vaccination is recommended. With some evidence that pneumococcal vaccinations are not as effective when administered after starting disease-modifying antirheumatic drugs (DMARDs), in particular methotrexate,^1^ guidance on when best to vaccinate, in relation to DMARDs, has become more consistent in recent years. Early European League Against Rheumatism guidelines (2011) only referred to B-cell depleting biological DMARDs, but more recent guidelines (2019)^2^ recommend vaccination prior to commencement of all DMARD types. Since 2011, British Society for Rheumatology (BSR) guidance advises vaccination prior to starting any DMARD.^3 4^ The aims of this study were to explore the timing of pneumococcal vaccination in patients with RA in relation to starting conventional synthetic DMARDs (csDMARDs) and examine whether this has changed over time.

This was a cross-sectional study using data from the Clinical Practice Research Datalink GOLD (UK primary care electronic health records). The study period was from 1 January 2000 to 31 December 2018. To be included, patients were required to (1) have a diagnosis of RA identified using a validated algorithm^5^; (2) be prescribed csDMARDs up to a maximum of 3 months prior to, or anytime after, RA diagnosis and (3) have received a pneumococcal vaccination up to a maximum of 5 years prior to, or anytime after, starting csDMARDs. For each patient, it was determined if vaccination was prior to starting csDMARDs.

**Figure 1:**
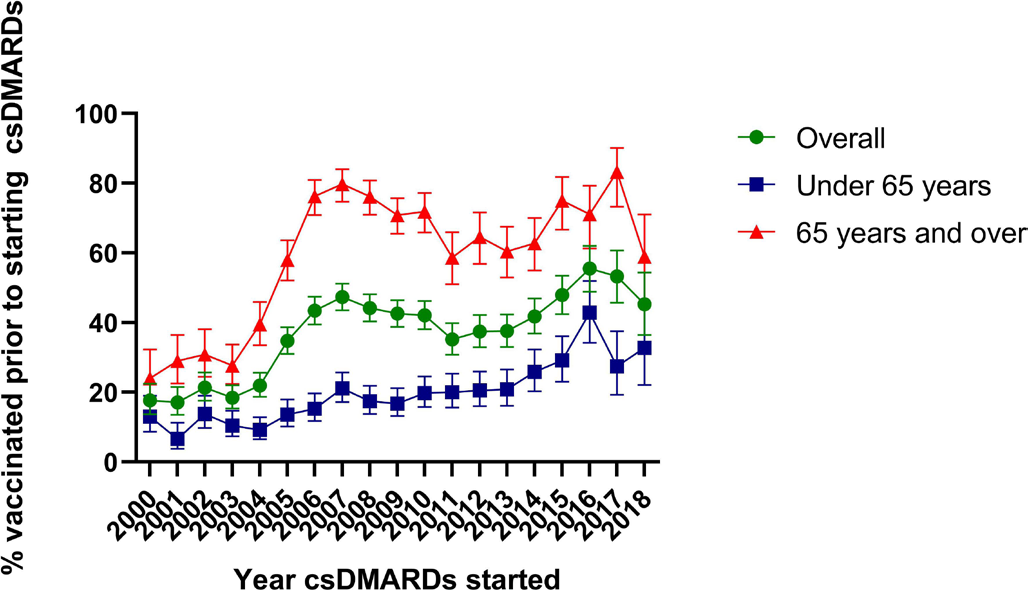
Timing of vaccination in relation to starting csDMARDs by year, overall and stratified by age. csDMARD, conventional synthetic disease-modifying anti-rheumatic drug.

Of 21 461 patients with RA who started csDMARDs within the study window, 8205 (38.2%) were vaccinated and met the inclusion criteria. Nearly half (44.3%, n=3633) were age ≥65 years, 66.4% (n=5445) were female and 26.7% (n=2188) had a diagnosis for another disease where vaccination is also recommended. Overall, 2997 (36.5%) patients were vaccinated prior to starting csDMARDs. When stratified by age, of those vaccinated prior to starting csDMARDs, 88% (n=1911/2170) of those age ≥65 years and 72% (n=596/827) of those <65 years, were vaccinated prior to RA diagnosis. The frequency of vaccination was higher in the first year after starting csDMARDs, with 1779 (21.7%) vaccinated compared to 833 (10.2%) in the year preceding (online supplementary figure 1). However, 1000 (12.2%) were vaccinated >3 years prior, and 1844 (22.5%) were vaccinated >3 years after starting csDMARDs. By calendar year, the proportion vaccinated prior to starting csDMARDs increased over time, with the greatest increases seen between 2003 and 2007 where the proportion increased from 18% to 47%. When stratified by age (65 years or over, when UK guidelines recommend vaccinating everyone against pneumococcus), the proportions vaccinated prior to starting csDMARDs were higher overall in the ≥65 years old age group. In those <65 years, the proportions rose more steadily over time from 13.0% in 2000 to 29.2% in 2015 (figure 1).

10.1136/annrheumdis-2020-217255.supp1 Supplementary data

This study has shown that, of patients with RA who received pneumococcal vaccinations, only around one-third of vaccinations occurred prior to starting csDMARDs. This is similar to a study in the USA where 41% were vaccinated prior to starting csDMARDs.^6^ There was evidence that commencement of csDMARDs prompts vaccination, however the peak in vaccination was in the year after starting csDMARDs. Vaccination prior to starting csDMARDs has increased through time with greater increases seen in those aged ≥65 years. A marked increase followed the 2003 change in national guidelines recommending all adults≥65 years should receive a pneumococcal vaccination. Indeed, most patients ≥65 years old vaccinated prior to starting csDMARDs were vaccinated prior to RA diagnosis. In those <65 years old, there were still increases over time, which was positive. Encouragingly, overall, there was also a steady increase from 2011, when BSR vaccination guidelines were published.^3^ Although the proportion decreases in 2018, further data are required to determine if this is a long-term trend. Given recent guidelines, we encourage rheumatologists to promote awareness of the importance of vaccinations prior to csDMARD initiation through timely communications to patients and primary care physicians.
