# Supplementary figures and images for: Pneumonia vaccination timing in relation to starting conventional synthetic disease-modifying antirheumatic drugs in patients with rheumatoid arthritis
Source: Ann Rheum Dis. 2020 Jun 26;79(12):1665–6. doi: 10.1136/annrheumdis-2020-217255 (PMC7677482; doi:10.1136/annrheumdis-2020-217255)

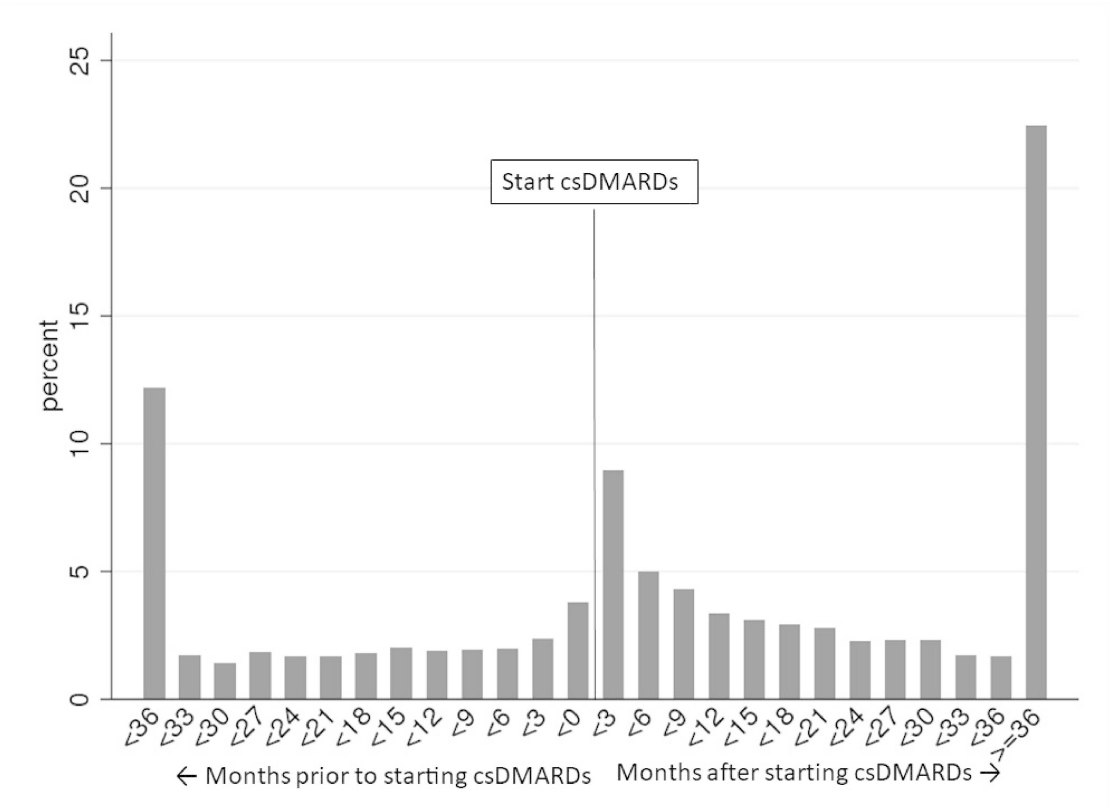

Supplement: Supplementary data [file annrheumdis-2020-217255supp001.pdf]
